# The impact of brand image on customer satisfaction and brand loyalty: A systematic literature review

**DOI:** 10.1016/j.heliyon.2024.e36254

**Published:** 2024-08-13

**Authors:** Abdul Haseeb Tahir, Muhammad Adnan, Zobia Saeed

**Affiliations:** a School of Economics and Management, University of Aden, Yemen; b Department of Psychology, University of Aden, Yemen

**Keywords:** Brand image, Brand trust, Foreign tourism, Brand loyalty, Service quality, Customer satisfaction

## Abstract

This literature review examines the impact of brand image on customer satisfaction and brand loyalty in the context of foreign tourism. Following a review of relevant literature, 13,302 articles were found for this study, including the keywords “brand image (BI)”, “customer satisfaction (CS)”, and “brand loyalty (BL)”. Considering the required inclusion and the quality of studies, we employed the rigorous PRISMA technique for comprehensive data synthesis and evaluated 79 articles for the final review. Our findings underscore the significant impact of brand image on shaping customer satisfaction and fostering brand loyalty within the foreign tourism sector. The study enriches the literature by incorporating self-congruity theory. In addition, factors like product quality, pricing, and advertising are identified as key determinants significantly influencing the proposed relationship.

## 1 Introduction

Companies dedicate their resources to building their brand image, including effort, time, and money. Brands decide on the style they should adopt and how customers feel when engaging with the brand, leading to a defined brand identity. This identity gives meaning to the brand as consumers associate with and learn about it [1]. Over the past few years, brands have become more in tune with market trends. Brand imagery, an essential component, significantly affects customers’ perceptions [2,3]. Brand image has become a significant part of branding. It refers to the ideas, beliefs, or perceptions about an entity [4]. In competitive environments, businesses provide high-quality services with exceptional brand images to achieve customer satisfaction and loyalty [5]. The brand image shows how the customer thinks about the brand and suggests how consumers perceive it, which is reflected in the associations they hold in their minds [6]. Literature also indicates that a brand image comprises a group of organized, meaningful perceptions [7].

A brand image is an asset that shapes customer beliefs about a brand [8]. The accounting dimensions of brand image, such as brand meaning, attributes, and associations, shape customer’s perceptions and influence their beliefs about the brand’s value [2]. Popular brands have a significant market presence, and their reputation plays an essential role in the customer’s mind [9,10]. The benefits of a brand image relate to five aspects: experience, symbolism, functionality, social, and appearance changes [11,12]. Customers learn about the product and brand through advertising, which enhances the brand image [13]. The image influences thoughts, feelings, and opinions and can be decisive in selecting competitors [14].

Literature suggests that customers buying many products indicate marketing success [15]. When creating an image for a product or service, one can select only a few ideas from the total impressions surrounding it. In the image creation process, these selected impressions will be refined and organised to shape the overall perception effectively [16,17]. A company’s market success is based on its capability to attract, satisfy, and retain customers by understanding their expectations. Literature suggests satisfaction occurs when customer feedback meets customer expectations [18]. This satisfaction has more chances to convert to loyalty. Satisfaction is a customer’s general attitude towards a service provider, or an emotional response to the difference between what customer expects and receives when a particular need, goal or desire is met [19]. In other words, the customer will be satisfied if the agreement meets the customer’s requirements [20].

Satisfaction is the emotional response that emerges after consumption from comparing expected performance with the experience [21]. Customer satisfaction results from perceived outcomes against consumer expectations [22]. Customer communication depends on satisfaction, as satisfied customers spread favorable reviews [23]. Satisfaction reflects the consumer’s attitude towards the service provider, indicating their emotional response to the difference between customers’ expectations and outcomes [24]. At the same time [25], state that acceptance, happiness, rel6axation, anticipation, and happiness can be interrelated with satisfaction.

In recent years, brand image association with the tourism sector has gained significant attention, including tourist facilities and cultural appeal [26]. Kim and Chung [27] and Jeong and Kim [28] examined the importance of building an emotional connection with the brand regarding brand destination, which ultimately builds customer trust, satisfaction, and confidence. According to Indelicato and Martín [29], there are five stages of customer trust: first is Loyalists (Consumers are happy and their faith in a particular brand is at its peak), second is Defectors (Consumers are unhappy and their confidence level in buy the product is also not satisfactory so, they will change their brand), third is Terrorists (The consumer’s satisfaction is below their standards and the level of trust is zero), fourth is Hostages (One company binds buyers because it is a monopoly strategy), and fifth is Mercenaries (The brand does not interfere with this type of customer, so at this stage, there is no concern about satisfaction or trust).

It is anticipated that the tourism market will experience substantial growth with international visitors, which is projected to increase from US$140 million to US$230 million by the end of 2026. This expansion will necessitate the development of additional tourism products and services to cater to the increasing demand [30]. Foreign tourism has gained significant attention globally, with visitors seeking adventure and experience [26]. Competition is increasing daily and becomes a big challenge, and to lead the competition, businesses must build a strong brand image among their customers to stay in the marketplace. To overcome this challenge, it is crucial to ensure customer satisfaction and create a positive impression of their brands, making customers loyal, satisfied, and happy to return. Prior literature suggests that a brand’s image is vital for customer satisfaction and loyalty [31]. However, their more profound understanding of tourism is still unclear.

In this study, we seek to explore this gap by conducting a systematic literature review (SLR) to investigate the impact of brands on customer satisfaction and brand loyalty in the context of the foreign tourism industry. Drawing upon the self-congruity theory [32,33], we aim to explore the relationship between brand image, customer satisfaction, and brand loyalty in the context of the foreign tourism industry.

The objectives of this SLR encompass several ways. First, it aims to explore the comprehensive association between brand image, customer satisfaction, and brand loyalty in foreign tourism. Second, self-congruity theory [32,33] investigates whether available products tailored to the needs of tourists improve brand loyalty in outbound tourism based on their awareness of brand image. Third, it assesses the factors influencing the relationship between brand image, customer satisfaction, and brand loyalty, including pricing, quality, and advertisement. By exploring these dynamics, our study contributes significantly to the literature and provides valuable insight into developing effective brand positioning and strategies in the foreign tourism industry.

## 2 Methodology

This section navigates the systematic approach undertaken to investigate the impact of brand image on customer satisfaction and brand loyalty in foreign tourism. For our study, we used a systematic approach. Contributors from various places connected online and crafted a road map for this SLR. We systematically identified and evaluated relevant literature, followed by data screening. After the screening, we extracted and synthesised the data using PRISMA 2020. To enhance the scalability, we identified and evaluated relevant literature data to develop a list of keywords, including a range of dimensions of our key variables such as brand image, brand loyalty, customer satisfaction, brand trust, service quality, brand reputation, brand advocacy, brand association, willingness to pay, brand engagement and attitude. We captured the multifaceted nature of our key variables. Fig. 3 presents a word cloud for visualising the overview of the reviewed literature. We employed a combination of keywords in both single and combined ways on two extensive databases, Web of Science (WOS) and Scopus. Data was screened and filtered using different Boolean operators on the search bars of each database to identify and analyse studies on brand image, customer satisfaction, brand loyalty, and related keywords. The data reviewed in this systematic literature review is composed through the secondary data collection method based on common themes and conceptual frameworks. Further, we evaluated measurement tools used in the studies included in our review for comprehensive assessment.

**Fig. 1:**
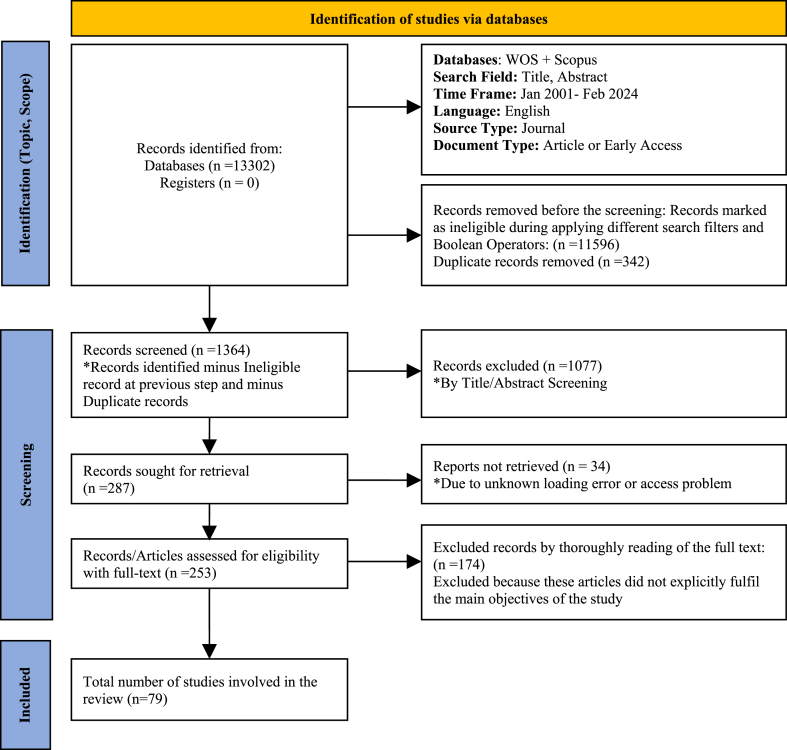
Prisma flow chart.

Source: Authors’ Constructed

**Fig. 2:**
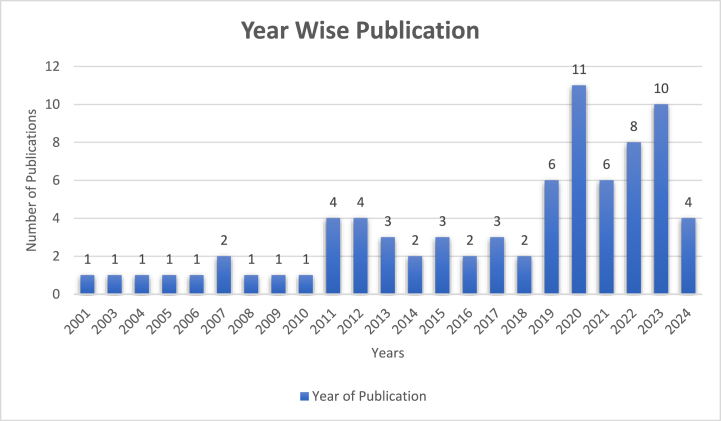
Number of included articles year-wise.

Source: Authors’ Constructed

13,302 articles were found from different databases using filters like the year of publication, e.g., 2001 to 2024. We selected only fully published and peer-reviewed articles with mentioned duration and criteria. Following manual screening, only articles containing relevant keywords in the title, abstract, or keyword section were selected from the initial pool of 13,302 articles. Several articles were filtered out during this process. Subsequently, duplicate and irrelevant articles were eliminated through manual screening. After reviewing the abstract, we selected 79 articles for our study by confirming their relevancy. For the systematic reviews, we employed Preferred Reporting Items and Meta-Analyses (PRISMA) [34] to elaborate the step-by-step process followed by Cheng, Rong, Wu, Zhou, Li, Li, Liang and Zhang [35]. We synthesised the data from various articles to provide a comprehensive overview of the relationship between brand image, customer satisfaction, and loyalty in foreign tourism. The methodological standard was employed to ensure our findings’ reliability and validity, thereby minimising the risk of bias. The whole process flow is given in Fig. 1. In total, 79 articles were reviewed, and the publication distribution by year is illustrated in Fig. 2.

### 2.1 PRISMA process Flow Chart

.

### 2.2 Word cloud

**Fig. 3:**
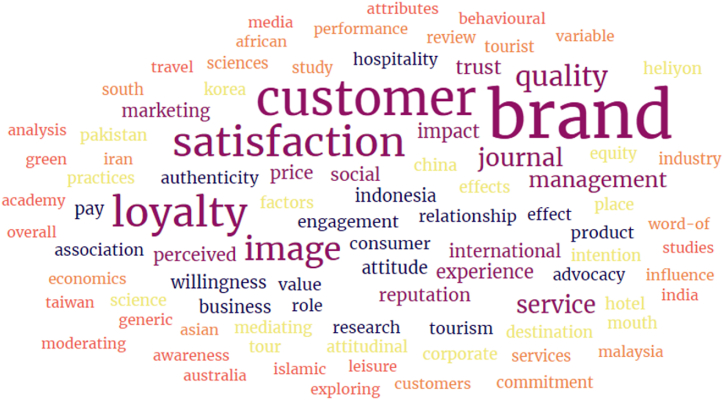
Word cloud.

Source: Authors’ Constructed

A word cloud visually represents text data that shows the frequency or importance of words within the chosen dataset. It’s also known as the co-occurrence of keywords, “tag cloud”, “text cloud”, or “weighted list”. This visual presentation lets one know which keyword was used most of the time in that selected data. We also did this to assess the possibility of replicability to understand the study and its replicability better. We used Table number 1 to evaluate the actual direction of screened articles, and we learned that customer satisfaction, brand loyalty, and brand image are frequently used, which means the studied article remained in discussion about these, as shown in Fig. 3.

## 3 Literature review

### 3.1 Relationship between brand image (BI) and customer satisfaction (CS)

Brand image (BI) plays a vital role in customer satisfaction (CS), and a strong brand image can satisfy customers because of their perceived quality and value [36]. The BI of a brand influences CS [37]. Marketers should design their products according to customers’ demands, and meeting customers’ expectations leads to satisfaction [23]. BI is a prominent and direct means of influencing CS [37]. A trusted and recognised brand image gives customers the confidence to use what the brand offers. Consequently, popular supermarkets also strive to create and represent strong brands consistently and transparently [38]. The match between expectations and experience determines consumer satisfaction with the product/service, the performance of the product/service, and the service provider’s service quality [39].

Bhatnagar and Nim [40] explored how CS helps with repeat business and argued that it should always be the mainstay of a service-oriented company. Also, one of the most effective ways to achieve this is by focusing on tangible things that attract customers. The four significant image benefits, experience, sociality, function, and appearance, have essential implications for satisfaction [41]. Focusing on BI and expertise can improve CS, as a strong brand image helps customers choose a company’s products or services [42].

BI positively impacts CS, which can be used as a benchmark for strengthening CS [31]. A well-known and accepted BI leads to CS [43]. Consumer satisfaction helps businesses recognize, identify, and anticipate brand goods, supporting BI conformation and growth [44]. Among the five image benefits, only experiential, psychological, practical image, and visual changes have a significant positive effect on satisfaction [11]. Since BI and SC are closely related, marketing managers must recognize the importance of creating a good brand identity to build consumer satisfaction or expect CS to establish loyalty [45]. Literature suggests that service companies’ managers should focus on more effective advertising methods to improve BI, ultimately leading to more incredible CS [46]. It is found that BI is a precursor to CS [47]. A successful BI positively impacts consumer loyalty and perceived quality, affecting how consumers view a business’s offerings and express satisfaction with those offerings for long-term enjoyment and sustainable benefit [48]. Consumers’ perception of alignment between their social introversion and the brand image positively affects their level of satisfaction with the brand [49]. Increased brand image and customer satisfaction can reduce advertising costs [38].

### 3.2 Relationship between brand image (BI) and brand loyalty (BL)

Literature indicates that a more excellent BI can lead to better customer service and increased brand loyalty (BL) [41]. Customers recommend leading brands due to their market image, performance, and symbolic values, highlighting the vital link between BI and BL. It is argued that brand awareness completely mediates BL, significantly shaping the strength of customer loyalty and overall brand equity [50]. BI directly and indirectly affects BL, while BI indirectly affects BL through CS [51]. Further, BI positively impacts brand extension, with loyalty mediating between the two [52]. Customers are more motivated to buy products if they match their personality, and a high product image enhances their social status.

### 3.3 Relationship between customer satisfaction (CS) and brand loyalty (BL)

CS and customer loyalty (CL) are valuable extensions of a positive BI, but there is some uncertainty about CL. The concept of loyalty can vary in evaluation and belief, with different researchers holding different opinions. While satisfying customers is essential, it is also crucial to ensure their happiness, so CL focuses on maintaining continuous customer loyalty [2]. BL helps reduce marketing expenses because loyal customers are more likely to continue purchasing the product [53]. Although CS does not guarantee customer repurchase, CL and retention remain essential in maintaining customer acceptance [54]. A satisfied customer is likely to purchase the same product again over time. However, if the satisfaction level is low or customers are not happy with the performance of products, they will stop using it [23,25].

A BI reflects three ideas in the customer’s mind: usefulness, social value, and showmanship. Product image choices often include appearance, features, objects, and the product’s role in consumer lives [3]. Literature suggests behavioural and attitudinal loyalty generates more future sales [55]. Factors such as price, brand reliability, brand awareness, and affiliation closely impact CS, and BL is strongly correlated with satisfaction, emphasizing their independence [56]. CS and BL may be influenced by the quality of information provided and the ease of the payment process [57]. CL is positively correlated with CS, with loyal customers expected to repurchase, recommend products to others, and remain loyal [36]. Consumer retention, however, does not reflect customer loyalty [58]. CS significantly affects CL indirectly [37].

Consumer expectations and the product’s benefits are crucial in satisfying customers [59]. Initially, a consumer is cognitively loyal, based solely on the brand’s feature values. True honesty and sincerity can arise from fulfilment based on brand success, leading to a more confident consumer mindset [60]. Demonstrating the connection between satisfaction and loyalty can be apparent by removing respondents who fall into the “region of indifference” [61]. A high degree of engagement and loyalty occurs when customers feel good about the relationship and inform others about the product or company [42]. CS and CL depend on the product’s nature, whether new, old, or familiar, and satisfaction with new products is more important than usual [2].

Previous research stated that customer perception of the product is essential in shaping consumer purchases. This perception influences customers’ attitudes toward the brand, affecting CS and CL [45,62]. Satisfied customers exhibit positive attitudes and communicate more positively about the brand [22,63]. It has been observed that BI helps create a clear and optimistic relationship between CS and CL. According to the literature [64], an effective marketing strategy emphasizes BI rather than satisfaction and desire for loyalty.

### 3.4 Relationship between brand image (BI), customer satisfaction (CS), and brand loyalty (BL)

BI, directly and indirectly, affects CL, indicating that brand image plays a significant role in creating CS and directly affects CL [64]. Businesses must concentrate on their primary customers, proactively generate high customer loyalty, meet consumer needs before competitors, and retain strong customer relations [12]. CS mediates between BI and BL [36]. BI, CS, and BL are interconnected. For long-term profitable relationships, all these factors need attention [37,65], especially in the religious sector, where people choose travel services based on good reputation [66].

BI significantly influences both CS and CL, with CS directly impacting CL [37]. Improvement in BI will lead to increased CS and BL [63]. Further, BI directly and indirectly impacts BL through the intermediary of CS [59]. To build a solid image in customers’ minds, businesses must focus on enhancing BI, CS, and CL [38]. Service-oriented businesses prioritize service quality to improve CS, BI, and BL [67,68].

Brand reputation (representing honesty and expertise) and satisfaction (summing up consumer purchase interactions by time) affect loyalty commitment (indicating mental adherence and a need to maintain future relationships) [69]. It is determined that CS fully mediates the correlation between BI and CL [70].

### 3.5 Relationship between price, customer satisfaction (CS), and brand loyalty (BL)

Price is a necessary commodity affecting the manufacturer’s profitability and the customer’s purchasing decisions. Four metrics characterizing price are affordability, product quality according to price, price competition, and price suitability for value [71]. Customer-specific prices are more competitive and offer similar quality at a lower cost than competitors, resulting in higher CS [72]. Customers are loyal because of the affordable price of products [36]. Literature suggests that the price factor directly influences CS, while price fairness positively influences CS, and uncertainty can adversely affect perceived market fairness [73]. Similarly, a price increase reduces CS [12]. Price positively and significantly impacts CS, indicating it can measure CS enhancements [31].

Slight price changes can lead to satisfied consumers, further building trust and associating the customers with the brand. However, if a product is discounted without meeting its required needs, it will fail to build confidence in the brand [59]. Literature highlights that BL is fundamental for achieving long-term profit, and cultivating loyal customers allows for greater flexibility in product price adjustments [38]. BI significantly influences BL, especially in retailing [74]. Customers can assess their satisfaction with a product or service based on different factors, including product features, price, customer service, or a combination of all these elements [42]. The price variable positively and substantially impacts customer loyalty [31]. Customer loyalty in attitude or behaviour may be influenced by product quality, brand popularity, store location, and price levels [75]. Consumer satisfaction with products and prices is crucial in deciding if a consumer will repurchase and recommend the product to others [10].

### 3.6 Relationship between service quality (SQ) on customer satisfaction (CS) and brand loyalty (BL)

Product service quality (SQ) and its variability may affect a company’s relationship with consumers, causing customers to respond to competitive proposals [76]. Better SQ helps to enhance CS and CL towards the brand [77,78]. CS is determined by product attributes, with performance measured against consumer pre-purchase expectations [23]. SQ is positively correlated with CS, so that quality can be used as an indicator of CS [31,79]. Since SQ is highly correlated, any quality change positively impacts CS [63]. Higher quality of a product increases CS, while innovation in SQ is used to enrich the SQ [67].

The interaction between SQ variables and BI strongly affects CS; key components of this interaction include responsiveness, empathy, and tangible aspects of the brand [43]. The perceived quality of service significantly impacts CS, with higher SQ producing higher CS [14]. SQ impacts CL indirectly through the mediation of CS [54]. The level of service directly or indirectly affects consumer loyalty, indicating that SQ plays a crucial role in creating both CS and CL. Moreover, SQ directly affects CL [64]. Better SQ will lead to increased BL [72]. Satisfied customers, resulting from high SQ, remain loyal to the brand [67]. BI can positively impact the perceived quality the market provides customers, thereby boosting CS and CL [80].

## 4 Results and discussion

This research examined how the brand image (BI) influences customer satisfaction (CS) and customer loyalty (CL), specifically for foreign tourism, as different articles were studied to explain this concept. The study explored different authors’ work and found that several variables (i.e., price, service, quality, advertisement) directly or indirectly influence BI, CS, and CL within this context of foreign tourism. The study found that BI positively impacts CS when marketers succeed in meeting customers’ expectations, which ultimately leads to CS. The finding of this SLR corroborates with the previous research in the same field [53,81,82]. The SLR also found that CS directly influences CL; increased CS leads to more incredible BL. This finding also extends the debate, which aligns with previous researchers who argued that CS and BL are strongly linked [39,56].

Furthermore, it has been found that price significantly affects foreign tourists because customers who are pleased with the pricing are likely to stay loyal to the brand [83]. Price affordability may also satisfy them and lead to BL [22,63]. The review study also found that minimal price changes can lead to CS, trust, and BL, as seen by earlier researchers [15,57,59]. Further, an important variable named “service quality” was explored during a systematic review, suggesting that service quality impacts CS and CL directly and indirectly. Providing higher service quality to tourists increases satisfaction, leading to BL [15,84,85]. During the review, the authors also observed specific gaps in the measurement and scalability of loyalty factors. This finding highlights the need to deepen our understanding of BL in the context of foreign tourism. Research also indicates that satisfied customers are likely to share positive word of mouth., which overcomes the advertisement cost, and this exploration is also supported by several authors [13,86]. The overall findings of this study suggest that BI has a direct and significant relationship with CS and BL in foreign tourism, underscoring its importance.

## 5 Conclusion and recommendations

This SLR underscores the significant impact of BI on CS and BL within the context of foreign tourism. As per self-congruity theory [33], the study found that BI significantly impacts CS and CL in the context of foreign tourism. We also identified key factors such as service quality, perceived value, brand trust, and emotional engagement that expanded our exploration of the relationship. Our findings suggest that customers naturally tend towards brands that have a good image in their minds. The study also highlights the role of branding in terms of quality, advertisement, and pricing, as it enhances the positive brand experience, loyalty, and emotional connection with customers [1]. The study also highlights implications for foreign tourism practitioners to develop effective marketing strategies that leverage brand image to enhance CS and CL. By recognising factors such as customer engagement, attitude, and relationship marketing, businesses can form more targeted strategies that will help improve customer experiences, CS, and BL.

Additionally, this SLR highlights that the success of a brand in the tourism sector depends on how the stakeholders align their brand with customers’ beliefs and interests, thereby creating a memorable brand experience. Researchers and practitioners must continue investigating how brands may employ technology to strengthen stakeholder relationships, leading to higher participation, advocacy, co-creation, and willingness for future purchases. They must focus on Corporate Social Responsibility (CSR), which provides greater trust and a stronger sense of connection among stakeholders with a brand [[87], [88], [89]]. This SLR also emphasizes advancing the understanding of BL and recommends a standardised measurement tool to explore the emerging dimension of loyalty. Moreover, to assess CS and CL, companies must evaluate their perceptions about products or services. Companies should determine whether satisfied customers are inclined to recommend the product to others or intend to make future purchases. Finally, to establish a successful brand, companies must prioritize the development of a positive BI to enhance CS and BL in an increasingly competitive marketplace.

## 6 Limitations and future research

The SLR focuses specifically on the impact of BI on CS and BL in the context of foreign tourism. This narrow scope may overlook valuable insights from other industries or contexts. We tried to cover all relevant literature, but selection bias may occur because this SLR analysed 79 articles, which may not represent the entire body of literature on the topic and could skew the findings. Variability in research methodologies, sample sizes, and data collection methods could impact the consistency of findings and may not be universally applicable beyond the specific context of foreign tourism. In addition, Cultural, geographical, and economic differences across regions may influence the relationship between BI, CS, and BL differently. Future research could compare the impact of BI on CS and CL across various industries to identify sector-specific differences because results may vary due to sector differences. Future research could conduct a longitudinal study and search for the long-term effects of BI on CS and CL, tracking changes over time and exploring potential causal relationships. Future research can also be done by combining quantitative and qualitative research methods, such as interviews or focus groups, offering a deeper understanding of the underlying mechanisms and customer insights driving the relationship between BI, CS, and CL.

Exploring the effects of cultural factors on the relationship between BI, CS, and CL can also be done because it can help tailor marketing strategies to diverse consumer segments in different regions. Moreover, future researchers can check the impact of CSR initiatives [88] and sustainability practices on BI, CS, and CL within the context of foreign tourism, considering growing consumer concerns about ethical consumption and environmental sustainability.

## Data availability statement

The data associated with this study has not been deposited into any publicly available repository because the selected articles were systematically reviewed and included in an article’s appendix with relevant details.

## Funding statement

The authors declare that no funds, grants, or other support were received during the preparation of this manuscript.

## Additional information

No additional information is available for this paper.

## Ethical approval

It is not applicable because “this work is a literature review and does not address the ethical considerations of animal, cell, and human experimentation”.

## Informed consent

Not applicable.

## CRediT authorship contribution statement

**Abdul Haseeb Tahir:** Methodology, Conceptualization. **Muhammad Adnan:** Writing – original draft, Data curation. **Zobia Saeed:** Writing – review & editing, Data curation.

## Declaration of competing interest

The authors declare that they have no known competing financial interests or personal relationships that could have appeared to influence the work reported in this paper.
