# Supplementary material for: The impact of brand image on customer satisfaction and brand loyalty: A systematic literature review
Source: Heliyon. 2024 Aug 13;10(16):e36254. doi: 10.1016/j.heliyon.2024.e36254 (PMC11378958; doi:10.1016/j.heliyon.2024.e36254)
Supplement: Multimedia component 1 [file mmc1.docx]

**The Impact of Brand Image on Customer Satisfaction and Brand Loyalty: A Systematic Literature Review**

**Appendix:**

**A –**

**Selected Articles with details for Systematic Literature Review**

| **Sr #** | **Name of Journal** | **Title of Article** | **Year** | **country/ selection/ context** | **Independent variable** | **Mediating variable** | **Moderating variable** | **Dependent variable** |
| --- | --- | --- | --- | --- | --- | --- | --- | --- |
| 1 | International Journal of Contemporary Hospitality Management | The Relationship Between Customer Loyalty and Customer Satisfaction | 2001 | USA | Customer Satisfaction | - | - | Customer Loyalty |
| 2 | Journal of Hospitality and Leisure Marketing | The Role of Customer Satisfaction and Image Gaining Customer Loyalty in the Hotel Industry | 2003 | Indonesia | Brand Image, Price, Performance | - | - | Customer Loyalty and Satisfaction |
| 3 | Journal of Service Marketing | Customer Satisfaction Should Not Be the Only Goal | 2004 | Australia | Advertisement, Brand Image | - | - | Customer Loyalty |
| 4 | Journal of Hospitality and Tourism Research | The Effects of Image Congruence on Customers Brand Loyalty in the Upper-Middle-Class Hotel Industry | 2005 | Missouri | Brand Image | Customer Satisfaction | - | Brand Loyalty |
| 5 | Academy of Marketing Science | The Importance of Customer Satisfaction vqin Relation to Customer Loyalty and Retention | 2006 | Malaysia | Customer Satisfaction | - | - | Loyalty, Retention |
| 6 | Asian Academy of Management Journal | The Effect of Brand Image on Overall Satisfaction and Loyalty Intention in the Context of Color Cosmetic | 2007 | Malaysia | Brand Image Benefits | Customer Satisfaction | - | Brand Loyalty |
| 7 | Journal of Marketing Research | The Influence of Price Fairness Customer Satisfaction | 2007 | USA | Perceived Vulnerability | Price offered | - | Satisfaction |
| 8 | University of Western Australia | The Role of Brand Credibility in Low Contact Relational Services, A Study in Two Industries | 2008 | Australia | Satisfaction, Loyalty Commitment, Continuance Commitment | Brand Credibility | - | Word-of-mouth, Switching Propensity |
| 9 | Journal of Technology Management in China | Exploring the Impact of Brand Image on Customer Loyalty and Commitment in China | 2009 | China | Brand Image | - | - | Customer Loyalty, Satisfaction, Commitment |
| 10 | European Journal of Interdisciplinary Studies | Product Or Brand? How Interrelationship between Customer Satisfaction and Customer Loyalty Work | 2010 | Vilnius | Product Type | Customer Satisfaction | - | Brand Loyalty |
| 11 | African Journal of Business Management | A Study on the Relations Between the Brand Image and Customer Satisfaction in Catering Businesses | 2011 | Taiwan | Brand Image | Core Values | - | Customer Satisfaction |
| 12 | African Journal of Business Management | Customer Satisfaction, Brand Trust and Variety Seeking as Determinants of Brand Loyalty | 2011 | South Africa | Perceived Quality, Customer Expectations | Perceived  Value, Customer  Satisfaction | - | Complaints, Loyalty |
| 13 | International Conference on Industrial Engineering and Engineering Management | Service Quality, Brand Image, and Price Fairness Impact on the Customer Satisfaction and Loyalty | 2011 | Taiwan | Brand Image | Customer Satisfaction | Service Quality, Price | Customer Loyalty |
| 14 | International Journal of Economics and Management Sciences | Impact of Brand Image, Trust, and Effect on Consumer Brand Extension Attitude: The Mediating Role of Brand Loyalty | 2011 | Pakistan | Brand Image And Trust | Loyalty | - | Brand Extension Attitude |
| 15 | African Journal of Business Management | The Investigation and Analysis Impact of Brand Image in Iran | 2012 | Iran | Innovation, Customer Satisfaction, Advertising | - | - | Brand Image |
| 16 | Journal of Business and Social Science | Impact of Brand Image, Service Quality, and Price on Customer Satisfaction in Pakistan Telecommunication Sector | 2012 | Pakistan | Brand Image | Service Quality | Price | Customer Satisfaction |
| 17 | Journal of Social and Development Sciences | Corporate Brand Image and Customer Satisfaction on Loyalty | 2012 | Taiwan | Brand Image | Customer Satisfaction | - | Brand Loyalty |
| 18 | Jurnal Manajemen Dan Kewirausahaan | The Impact of Brand Image, Customer Loyalty with Customer Satisfaction as A Mediator in McDonald’s | 2012 | Indonesia | Brand Image | Customer Satisfaction | - | Customer Loyalty |
| 19 | European Journal of Business and Management | The Impact of Bank Brand Image on Customer Satisfaction and Loyalty | 2013 | Kenya | Brand Image | Service | - | Customer Satisfaction/ Loyalty |
| 20 | Journal of Business and Retail Management Research | The Impact of Corporate Image and Reputation on Service Quality, Customer Satisfaction and Customer Loyalty | 2013 | Egypt | Brand Image | Quality | Customer Satisfaction | Customer Loyalty |
| 21 | World Applied Sciences Journal | The Relationship Among Brand Experience, Brand Image, and Customer Satisfaction of Facebook Users in Indonesia | 2013 | Malaysia | Brand Experience | Brand Satisfaction | - | Brand Image |
| 22 | Journal of Social Science Research | Effect of Brand Image on Customer Satisfaction & Loyalty Intention | 2014 | India | Brand Image | Product Or Service, Salesperson, Store | - | Customer Satisfaction |
| 23 | Middle-East Journal of Scientific Research | The Impact of Service Quality on Customer Satisfaction, Customer Loyalty, and Brand Image | 2014 | Pakistan | High Quality | Customer Satisfaction, Brand Image | - | Brand Loyalty |
| 24 | Asian Journal of Management Sciences | Interaction Effect of Perceived Service Quality and Brand Image on Customer Satisfaction | 2015 | Sudan | Brand Image | Service Quality | - | Customer Satisfaction |
| 25 | Business Management Dynamics | Mediating Roles of Customer Satisfaction and Customer Trust in Building Brand Loyalty. | 2015 | Pakistan | Brand Image Sale Promotion | Customer Satisfaction, Trust | - | Brand Loyalty |
| 26 | International Journal of Social Sciences and Management | The Effects of Brand Image on Customer Satisfaction and Loyalty | 2015 | London | Brand Image | - | - | Customer Satisfaction |
| 27 | Journal of Hospitality & Leisure Marketing | The Impact of Brand Image on Customer Satisfaction in Major Supermarkets | 2015 | Kenya | Brand Image | - | Product Performance | Customer Satisfaction |
| 28 | Advanced Social Humanities and Management | The Branding: A Study of Brand Image, Brand Associations and Reputation | 2016 | Iran | Brand Image | - | - | Brand Trust |
| 29 | Mediterranean Journal of Social Sciences | Impact of Service Quality, Price, and Brand on Loyalty with the Mediation of Customer Satisfaction | 2016 | Indonesia | Brand Image, Service, Price | Customer Satisfaction | - | Brand Loyalty |
| 30 | International Journal of Health Care Quality Assurance | Brand Trust and Image: Effects on Customer Satisfaction | 2017 | Iran | Brand Image | - | - | Customer Satisfaction |
| 31 | International Journal of Innovation and Applied Studies | Brand Loyalty Brand Image and Brand Equity: The Mediating Role of Brand Awareness | 2017 | Pakistan | Brand Promise Quality | Brand Awareness | - | Brand Image, Brand Equity, Brand Loyalty |
| 32 | UEU-Master-6266-International Journal | The Impact of Service Quality and Brand Image on Customer Loyalty Mediated by Customer Satisfaction | 2017 | Indonesia | Brand Image Service Quality | Customer Satisfaction | - | Customer Loyalty |
| 33 | IFAC-Paper Online | Switching Cost, Corporate Image, and Product Quality Effect on Customer Loyalty | 2018 | Balkans | Cost | - | Quality | Brand Loyalty |
| 34 | International Review of Management and Marketing | Product Quality, Brand Image, and Pricing to Improve Satisfaction | 2018 | Indonesia | Brand Image | Price-Quality | - | Customer Satisfaction |
| 35 | Academy of Strategic Management Journal | The Parcelling of Loyalty: Brand Quality, Brand Affect, and Brand Trust Effect on Attitudinal Loyalty and Behavioral Loyalty | 2019 | Indonesia | Brand Quality, Brand Affect, And Brand Trust | - | - | Attitudinal Loyalty and Behavioural Loyalty |
| 36 | International Review of Management and Marketing | Effect of Brand Image, Quality, and Price on Customer Satisfaction and Implications for Customer Loyalty PT Strait Liner Express in Jakarta | 2019 | Jakarta | Brand Image | - | Quality | Customer Satisfaction |
| 37 | Journal of Retailing and Consumer Services | Modelling the Relationship Between Hotel Perceived Value, Customer Satisfaction, and Customer Loyalty | 2019 | United Arab Emirates | Customer Satisfaction | Hotel Perceived Value Dimensions | - | Customer  Loyalty |
| 38 | Prabandhan: Indian Journal of Management | Impact of Housekeeping Services and Practices on Customer Satisfaction and Repeat Business | 2019 | India | Housekeeping Services and Practices | Customer Satisfaction | - | Repeat Business |
| 39 | Journal of Travel Research | Visitors’ Place Attachment and Destination Loyalty: Examining the Roles of Emotional Solidarity and Perceived Safety | 2019 | India | Place Dependence, Place Identity | Welcoming Nature, Emotional Closeness, Sympathetic Understanding | Perceived Safety | Destination Loyalty |
| 40 | Heliyon | The Impact of E-Service Quality and Customer Satisfaction on Customer Behavior in Online Shopping | 2019 | Indonesia | Website Design, Customer Service, Security/Privacy, Fulfilment | Overall E-Service Quality, Customer Satisfaction, Customer Trust, | - | Purchase Intention, Word of Mouth, Site Revisit |
| 41 | International Journal of Research in Business and Social Science | Effect of Customer Loyalty Program on Customer Satisfaction and Its Impact on Customer Loyalty | 2020 | Indonesia | Membership Card, Discount Promo. | Customer Satisfaction | - | Customer Loyalty |
| 42 | Journal of Business & Industrial Marketing | Goods and Services Related Brand Image and B2B Customer Loyalty | 2020 | India | Brand Image | Satisfaction | Construal Level | Brand Loyalty |
| 43 | Management Science Letters | Customer Satisfaction as A Mediation Between Micro Banking Image, Customer Relationship and Customer Loyalty | 2020 | Canada | Micro Banking Image, Customer Relationship | Customer Satisfaction, | - | Customer Loyalty |
| 44 | Asia Pacific Journal of Marketing and Logistics | A Study Of Event Quality, Destination Image, Perceived Value, Tourist Satisfaction, And Destination Loyalty Among Sport Tourists | 2020 | South Korea | Event Quality | Destination Image, Tourist Satisfaction, Perceived Value | - | Destination Loyalty |
| 45 | The Journal of Asian Finance, Economics and Business | The Relationship Between Brand Authenticity, Brand Equity and Customer Satisfaction | 2020 | Vietnam, | Brand Authenticity | Brand Equity | - | Customer Satisfaction |
| 46 | The TQM Journal | The Effect of Experience Quality on Customer Perceived Value and Customer Satisfaction and Its Impact on Customer Loyalty | 2020 | Indonesia | Experience Quality | Customer Perceived Value, Customer Satisfaction | - | Customer Loyalty |
| 47 | Journal of Travel Research | Consumption Authenticity in the Accommodations industry: the Keys to Brand Love and Brand Loyalty for Hotels and Airbnb | 2020 | Micro-Task Crowdsourcing Platform Amazon Mechanical Turk | Brand Authenticity, Existential Authenticity, Intrapersonal Authenticity | Brand Love | - | Brand Loyalty |
| 48 | Tourism Management | Will You Miss Me If I Am Leaving? Unexpected Market Withdrawal of Norwegian Joy and Customer Satisfaction | 2020 | Norwegian Joy and Quantum of The Seas Cruises From Ctrip Website | Customer Expectation, Customer Experience | Customer Satisfaction | - | Customers Continuance Intention And Loyalty |
| 49 | Tourism Management | Understanding the Dynamics of the Quality of Airline Service Attributes: Satisfiers and Dissatisfiers | 2020 | Consumer Review Websites, Namely, Trip advisor, | Quality of Airline Service Attributes | - | - | Overall Satisfaction |
| 50 | Tourism Management | Discovering the Perceived Attributes of CBT Destination Travellers in South Korea: A Mixed Method Approach | 2020 | Korea | Community-Based Tourism (CBT) Destination | Place Identity, Place Dependence | Awareness of Community Benefits of CBT | Destination Loyalty |
| 51 | Heliyon | Generic and Islamic Attributes for Non-Muslim Majority Destinations: Application of the Three-Factor Theory of Customer Satisfaction | 2020 | Tokyo  Seoul  Singapore  Bangkok  Hong Kong  Osaka  London  Kyoto  Other | Generic Basic Factors, Islamic Basic Factors, Generic Performance Factors, Islamic Performance Factors, Generic Excitement Factors, Islamic Excitement Factors, | Tourist Satisfaction, | - | Behavioural Loyalty, Attitudinal Loyalty |
| 52 | American International Journal of Business Management | The Influence of Product Quality, Brand Image, and Brand Trust on Customer Satisfaction and Loyalty | 2021 | Indonesia | Product Quality | Brand Image, Brand Trust, Customer Satisfaction | - | Customer Loyalty |
| 53 | Journal of Asian Finance, Economics and Business | Relationships Between Service Quality, Brand Image, Customer Satisfaction, and Customer Loyalty | 2021 | Vietnam | Service Quality | Brand Image | - | Customer Satisfaction, Brand Loyalty |
| 54 | Journal of Economics, Finance and Management Studies | Impact of Price, Product Quality, and Promotion on Consumer Satisfaction in Cosmetics and Skincare | 2021 | South Korea | Price, Product Quality, Promotion | - | - | Customer Satisfaction |
| 55 | International Journal of Retail & Distribution Management | Unpacking Brand Loyalty In Retailing: a Three-Dimensional Approach to Customer–Brand Relationships | 2021 | France | Hedonic Image, Functional Image, Symbolic Image | Brand Attachment, Brand Trust, Brand Identification | - | Affective loyalty, Cognitive loyalty, Normative loyalty |
| 56 | Tourism Management | This Must Be the Place: A Destination-Loyalty Model for Extreme Sporting Events | 2021 | France | Sensation-Seeking | Self-Enhancement, Place Attachment | Event Authenticity | Destination Loyalty |
| 57 | Heliyon | How Brand Attitude, Brand Quality, and Brand Value Affect Thai Canned Tuna Consumer Brand Loyalty | 2021 | Thailand | Brand Attitude | Brand Quality, Brand Value |  | Brand Loyalty |
| 58 | Cornell Hospitality Quarterly | Measuring Customer Satisfaction and Hotel Efficiency Analysis: An Approach Based on Data Envelopment Analysis | 2022 | South Korea | Service Productivity | - | - | Customer Satisfaction |
| 59 | International Journal of Data and Network Science | The Role of Social Media Marketing and Brand Image on Smartphone Purchase Intention | 2022 | Indonesia | Social Media Marketing | Brand Image | - | Purchase Intention |
| 60 | International Journal of Social Science | Service Quality, Customer Value, and Price to Consumer Satisfaction at Kopi Kenangan Coffee Shop | 2022 | Indonesia | Service Quality, Customer Value, And Price | - | - | Consumer Satisfaction |
| 61 | Journal of Relationship Marketing | The Impact of Customer Relationship Management and Company Reputation on Customer Loyalty: the Mediating Role of Customer Satisfaction | 2022 | Pakistan | Customer Relationship Management, Company Reputation | Customer Satisfaction | - | Customer Loyalty |
| 62 | Public Management Review | Internal Brand Management in the Public Sector: The Effects of Internal Communication, Organisational Practices, and PSM on Employees’ Brand Perceptions | 2022 | Sweden | Public Service Motivation | Value Congruence in Internal Communication and Positive Organizational Practices | - | Brand Identification, Brand Pride, Brand Commitment |
| 63 | Tourism Management | Development and Validation of A Casino Service Quality Scale: A Holistic Approach | 2022 | U.S. gaming market | Gaming Service, Restaurant Service, Hotel Service, Cleanliness | CASQUAL | - | Satisfaction, Loyalty |
| 64 | International Journal of Hospitality Management | The Effects of Consumer Brand Authenticity, Brand Image, and Age on Brand Loyalty in Time-Honored Restaurants: Findings from SEM and fsQCA | 2022 | China | True-To-Fact Authenticity, True-To-Ideal Authenticity | True-To-Self Authenticity, Brand Image, | Consumer Age | Brand Loyalty |
| 65 | Heliyon | Modelling the Significance of Social Media Marketing Activities, Brand Equity and Loyalty to Predict Consumers’ Willingness to Pay Premium Price for Portable Tech Gadgets | 2022 | Malaysian | Entertainment, Interactivity, Trendiness, Customization, Electronic Word-of-Mouth | Brand awareness, Brand Image, Brand Loyalty | - | Willingness To Pay Premium Price |
| 66 | African Journal of Hospitality, Tourism and Leisure | Increase Brand Loyalty Through Customer Satisfaction at Restaurants | 2023 | Indonesian | Product Quality, Service Quality | Customer Satisfaction | - | Brand Loyalty |
| 67 | Behavioral Science | The Mediating Role of Customer Satisfaction Between Antecedent Factors and Brand Loyalty for the Shopee Application | 2023 | Malaysia | Price of Delivery, Information Quality, Accuracy of Delivery, Ease of payment, Security of Payment | Customer Satisfaction | - | Brand Loyalty |
| 68 | Journal of Hospitality & Tourism Research | Marketing A Destination Brand Image to Muslim Tourists: Does Accessibility to Cultural Needs Matter in Developing Brand Loyalty? | 2023 | New Zealand | Tourism Host Brand Image, Accessibility to cultural needs | Customer Satisfaction | Religiosity | Brand Loyalty |
| 69 | SAGE Open | The Influence of Social Marketing Drives on Brand Loyalty Via Customer Satisfaction as A Mediating Factor in Travel and Tourism Offices | 2023 | Jordan | Social Media Marketing Factors | Customer Satisfaction | - | Brand Loyalty |
| 70 | International Journal of Retail & Distribution Management | Engaging The Customer With Augmented Reality and Employee Services to Enhance Equity and Loyalty | 2023 | China | Service Experience with Employees, Perceived Interactivity, Service Experience with AR | Customer Satisfaction | - | Perceived Customer  Equity, Customer Loyalty |
| 71 | Uncertain Supply Chain Management | The Effects of Customer Satisfaction, Perceived Service Quality, Perceived Value, and Brand Image  on Customer Loyalty | 2023 | Indonesian | Customer Satisfaction, Perceived Service Quality, Perceived Value, And Brand Image | - | - | Customer Loyalty |
| 72 | Journal of Travel Research | From tourist Experience to Satisfaction and Loyalty: Exploring the Role of a Sense of Well-Being | 2023 | Portugal | Tourist Experience | Tourist Satisfaction | - | Tourist Loyalty |
| 73 | Heliyon | Positive Effects of Green Practices on Consumers’ Satisfaction, Loyalty, Word-of-Mouth, and Willingness to Pay | 2023 | Chile | Green Practices Perception | Satisfaction, Willingness to Pay | - | Loyalty, Word-of-Mouth |
| 74 | Heliyon | Investigating the Role of Utilitarian and Hedonic Goals in Characterizing Customer Loyalty in E-Marketplaces | 2023 | Indonesia | Perceived Service Quality, System Personalization | Customer satisfaction, Customers’ Perceived Enjoyment, Customers’ Extent of Confirmation | - | Customer Loyalty |
| 75 | Heliyon | Does Corporate Social Responsibility Result in Better Hotel Guest Attitudinal and Behavioral Loyalty? | 2023 | Morocco | Corporate Social Responsibility | Customer Satisfaction, Trust, Customer-Company Identification | - | Customer Behavioural Loyalty, Customer Attitudinal Loyalty |
| 76 | International Journal of Hospitality Management | Unveiling the Dynamics Between Consumer Brand Engagement, Experience, and Relationship Quality towards Luxury Hotel Brands: Moderating investigation of Brand Reputation | 2024 | India | Service Environment | Consumer Brand Engagement, Consumer Brand Experience | Brand Reputation | Brand Equity, Brand Relationship Quality |
| 77 | Heliyon | Corporate Social Responsibility’s Impact on Passenger Loyalty and Satisfaction in the Chinese Airport industry: the Moderating Role of Green HRM | 2024 | China | Corporate Social Responsibility | Passenger Satisfaction | Green Human Resource Management | Passenger Loyalty |
| 78 | Heliyon | Optimising Brand Loyalty Through User-Centric Product Package Design: A Study of User Experience in the Dairy Industry | 2024 | South Korea | Sensory Experience, Emotional Experience, Action Experience, Thinking Experience | Brand Image, Brand Trust | - | Brand Loyalty |
| 79 | Corporate Reputation Review | Examining the Impact of Sensory Brand Experience on Brand Loyalty | 2024 | China | Sensory Brand Experience, | Customer satisfaction, brand attachment, customer love marks | Employee empathy | Attitudinal loyalty, behavioural loyalty |
